# Identification and development of long non‐coding RNA‐associated regulatory network in colorectal cancer

**DOI:** 10.1111/jcmm.14395

**Published:** 2019-05-29

**Authors:** Hongda Pan, Jingxin Pan, Shibo Song, Lei Ji, Hong Lv, Zhangru Yang

**Affiliations:** ^1^ Department of Gastric Surgery Fudan University Shanghai Cancer Center Shanghai China; ^2^ Department of Hematology The Second Attached Hospital of Fujian Medical University Quanzhou China; ^3^ Department of Oncology Shanghai Medical College, Fudan University Shanghai China; ^4^ Department of Gastrointestinal Surgery Beijing Hospital Beijing China

**Keywords:** bioinformatics analysis, colorectal cancer, competing endogenous RNA, long non‐coding RNA, prognostic biomarker, The Cancer Genome Atlas

## Abstract

Colorectal cancer (CRC) is one of the leading causes of cancer‐associated death globally. Long non‐coding RNAs (lncRNAs) have been identified as micro RNA (miRNA) sponges in a competing endogenous RNA (ceRNA) network and are involved in the regulation of mRNA expression. This study aims to construct a lncRNA‐associated ceRNA network and investigate the prognostic biomarkers in CRC. A total of 38 differentially expressed (DE) lncRNAs, 23 DEmiRNAs and 27 DEmRNAs were identified by analysing the expression profiles of CRC obtained from The Cancer Genome Atlas (TCGA). These RNAs were chosen to develop a ceRNA regulatory network of CRC, which comprised 125 edges. Survival analysis showed that four lncRNAs, six miRNAs and five mRNAs were significantly associated with overall survival. A potential regulatory axis of ADAMTS9‐AS2/miR‐32/PHLPP2 was identified from the network. Experimental validation was performed using clinical samples by quantitative real‐time PCR (qRT‐PCR), which showed that expression of the genes in the axis was associated with clinicopathological features and the correlation among them perfectly conformed to the ‘ceRNA theory’. Overexpression of ADAMTS9‐AS2 in colon cancer cell lines significantly inhibited the miR‐32 expression and promoted PHLPP2 expression, while ADAMTS9‐AS2 knockdown had the opposite effects. The constructed novel ceRNA network may provide a comprehensive understanding of the mechanisms of CRC carcinogenesis. The ADAMTS9‐AS2/miR‐32/PHLPP2 regulatory axis may serve as a potential therapeutic target for CRC.

## 1 INTRODUCTION

Colorectal cancer is the third most common malignancy and the fourth leading cause of death globally and about 1 400 000 new cases and 700 000 deaths occurred in 2012.^1^ According to the data of Chinese National Cancer Center, CRC is the fourth most common cancer in women and the fifth in man, China suffered 376 300 new cases and 191 000 deaths of CRC in 2015.^2^

Although researchers have made great efforts to elucidate the underlying tumourigenesis mechanism of the development of colorectal cancer, much of it is still unclear. Therefore, it is critical to explore the regulatory mechanisms of colorectal tumourigenesis to promote identification of promising diagnostic biomarkers as well as development of optimal therapeutic strategies.

Long non‐coding RNAs (lncRNA) are recently discovered type of non‐coding RNAs (ncRNA) with transcript more than 200 bp in length.^3^ lncRNAs have been recognized to play important roles in tumourigenesis via regulating the expression of genes. Increasing number of evidence has illustrated that lncRNAs expression profiling may become useful tool for the diagnosis of cancers.^4^, ^5^ Although a great amount of lncRNAs has been annotated, more efforts are needed to identify the function of them.^6^

The hypothesis of competing endogenous RNA (ceRNA) described that by sharing miRNA response elements (MREs), lncRNAs and mRNAs compete for binding to miRNAs, which exert the role of regulating each other's expression^7^ and affect the tumourigenesis and development of tumours.^8^, ^9^

For the past few years, the widespread application of microarray and high throughput sequencing has greatly promoted the process of development of useful biomarkers for cancer diagnosis and management. Thanks to the publicly available cancer genomic databases, such as The Cancer Genome Atlas (TCGA), the comprehensive bioinformatics analysis has been employed in cancer research, which facilitates the discovery of a vast range of valuable biological information. Based on the ceRNA theory, researchers tried to construct lncRNA‐miRNA‐mRNA ceRNA networks in several kinds of malignance.^10^, ^11^, ^12^, ^13^, ^14^ However, the data mining and analysis methods used in these reports were discrepant and the results were ambiguous and unconvincing. Out study first analysed the dysregulated expression of mRNA, lncRNA and miRNA between CRC tumour and normal tissues from TCGA database. Subsequently, the lncRNA‐mediated ceRNA network was developed by bioinformatics prediction and correlation analysis. The RNAs functioning as prognostic biomarkers for patients with CRC were further identified by survival analysis. This study was conducted to investigate how the lncRNAs regulate target genes in the ceRNA regulatory network in CRC and predict novel lncRNAs as therapeutic targets and potential diagnostic biomarkers.

## 2 MATERIALS AND METHODS

### 2.1 Patients and TCGA data retrieval

By using the GDC Data Transfer Tool, the level 3 transcriptome sequencing data, miRNAseq data and corresponding clinical information of those patients were downloaded from TCGA database (up to August 23, 2018). The TCGA‐COAD dataset contains 458 colon cancer samples and 41 normal samples for lncRNA and mRNA sequencing and 439 colon cancer samples and eight normal samples for miRNA sequencing. TCGA‐READ dataset contains 167 rectal cancer samples and 10 normal samples for lncRNA and mRNA sequencing and 180 rectal cancer samples and three normal samples for miRNA sequencing (Table S1). Replicated cases and cases without complete transcriptome profiling data were excluded. The expression profiling was performed by Illumina HiSeq RNASeq and Illumina HiSeq_miRNASeq platforms respectively. mRNAs and lncRNAs were identified and annotated by using the Ensembl database,^15^ RNAs which were not annotated in the database were excluded. This study was performed following the TCGA publication guidelines. As the data were all retrieved from TCGA, approval from a local Ethics Committee were unnecessary.

### 2.2 Identification of differentially expressed lncRNAs, mRNAs and miRNAs

The lncRNA, mRNA and miRNA expression data of tumour and normal samples were merged for COAD and READ respectively. Rows of RNA data with no expression or a mean count of ≤1 were deleted. To obtain the differentially expressed lncRNAs, mRNAs and miRNAs between normal tissue and CRC, the count data were processed with the Bioconductor package edgeR^16^ in R software. All RNA expression levels were standardized to the sample mean. The P value was corrected with a false discovery rate (FDR). Fold changes of expression levels (log2 absolute) ≥ 2 and FDR < 0.01 were considered as a statistically significant difference. Venn diagrams were made to select intersected RNAs that differentially expressed in both COAD and READ databases.

### 2.3 Functional enrichment analysis

To elucidate potential biological processes and to explore promising signalling pathways associated with the differentially expressed mRNAs (DEmRNAs), we conducted the Kyoto Encyclopedia of Genes and Genomes (KEGG) pathway enrichment analysis and the gene ontology (GO) enrichment analysis using the Database for Annotation, Visualization and Integrated Discovery (DAVID, version 6.8). The biological processes and pathways with *P* < 0.05 were considered as significant functional categories.

### 2.4 Development of a ceRNA network

**Figure 1:**
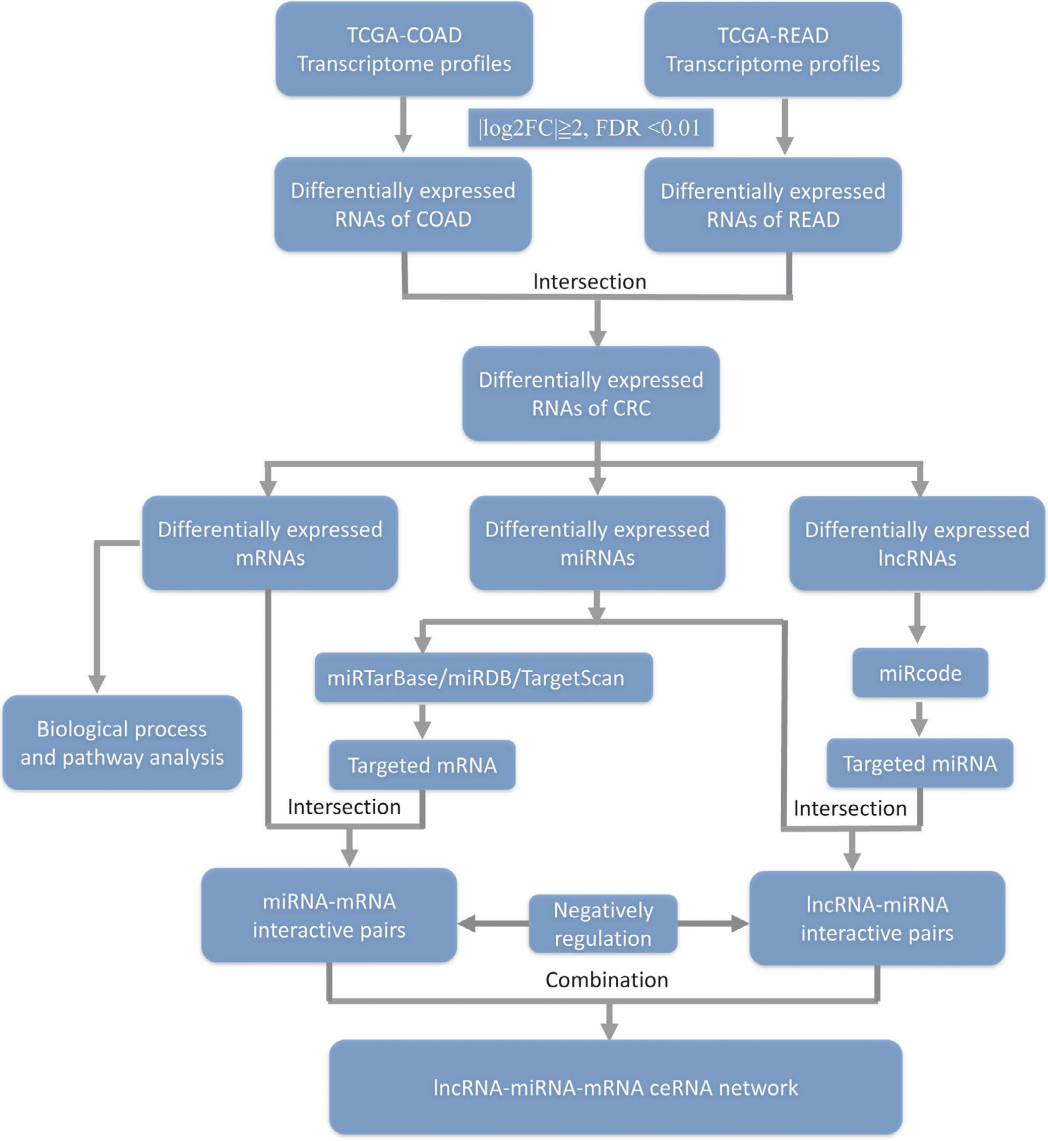
Flow chart of comprehensive bioinformatics analysis in the construction of competing endogenous RNA (ceRNA) regulatory network

The ceRNA network of CRC was then developed step‐by‐step according to the following procedures. Firstly, the file of potential interactions of lncRNA‐miRNA was downloaded from StarBase V3.0^17^ and miRcode.^18^ StarBase provides the most comprehensive CLIP‐Seq experimentally supported miRNA‐mRNA and miRNA‐lncRNA interaction networks to date. About 10 000 ceRNA pairs from CLIP‐supported miRNA target sites were identified in StarBase. MiRcode provides ‘whole transcriptome’ human microRNA target predictions based on the comprehensive GENCODE gene annotation, including >10 000 long non‐coding RNA genes. We filtered out the miRNAs that were not differentially expressed between tumour tissues and normal tissues. Next, three highly reliable online miRNA reference databases, miRTarBase,^19^ miRDB,^20^ and TargetScan,^21^ were used to retrieve experimentally validated or predictive miRNA targeted mRNAs. To improve the reliability of prediction, only the mRNAs presented in all three databases were defined as the miRNA‐targeted mRNAs. The targeted mRNAs were further compared with differentially expressed RNAs that identified between tumour tissues and normal tissues, the intersected mRNAs were retained to develop the ceRNA network. The ceRNA network was constructed and visualized by Cytoscape v3.6.1.^22^ Figure 1 shows a flow chart for the development of the ceRNA network. For the lncRNAs, mRNAs and miRNAs included in the ceRNA network of CRC, we generated heat maps using the ‘pheatmap’ packages in the r software. Key lncRNA was identified by calculating the degree of edges between lncRNA nodes and first stage miRNA nodes in the network. The expanded secondary miRNA‐mRNA interactomes were collected.

### 2.5 Clinical samples

A total of 50 primary CRC tissues and adjacent normal tissues were collected from Department of Gastrointestinal Surgery, Beijing Hospital. All tissues were frozen immediately in liquid nitrogen after surgical excision and stored at −80℃. Clinicopathological information was retrieved from the hospital database. Written informed consent was obtained from all patients and the study was approved by the ethics committee.

### 2.6 Cell culture and transfection

Human colon epithelial cell line (NCM460) and colon cancer cell lines (HT29, SW480 and SW620) were obtained from American Type Culture Collection (ATCC, Manassas). All cells were cultured in Dulbecco's modified Eagle's medium (DMEM) supplemented with 10% foetal bovine serum (FBS) in 5% CO_2_ atmosphere at 37°C. pcDNA3.1‐ADAMTS9‐AS2 and si‐ADAMTS9‐AS2 vectors were synthesized by Genechem (Shanghai, China) and then were transfected into CRC cells using Lipofectamine 3000 (Invitrogen).

### 2.7 Quantitative real‐time PCR

The expression levels of the key genes of the ceRNA regulatory axis were measured by quantitative real‐time PCR (qRT‐PCR). Total RNAs isolated from CRC tissues and cell lines by using TRIzol reagent (Invitrogen) and reverse transcription was performed with a Prime Script RT reagent kit (Takara Biotechnology, China). qRT‐PCR reactions were carried out on Applied Biosystems 7500 Real‐time PCR Systems (Thermo Fisher Scientific). Small RNA RNU6 (U6) was used as endogenous control to normalize miRNA, while GAPDH was used for lncRNA and mRNA expression. The relative expression level of the target RNA was calculated by 2^−ΔΔCt^.

### 2.8 Survival and statistical analysis

Survival analyses for all RNAs in the ceRNA network were performed by using the ‘survival’ package in r software. Kaplan‐Meier curve analysis with log‐rank test was performed for comparison of the survival differences between groups. Co‐expression analysis on the expression levels of RNAs was carried out by Pearson's correlation test. A *P < *0.05 was considered statistically significant.

## 3 RESULTS

### 3.1 Differentially expressed RNAs in CRC

Compared with normal tissue samples, a total of 920 differentially expressed lncRNAs, 2045 mRNAs and 249 miRNAs were identified in COAD samples. Meanwhile, 744 differentially expressed lncRNAs, 1998 mRNAs and 227 miRNAs were identified in READ samples. After intersection, 503 differentially expressed lncRNAs, 1419 mRNAs and 188 miRNAs were identified as cancer‐specific RNAs for CRC.

Of these, 313 (62.2%) lncRNAs, 632 (44.5%) mRNAs and 108 (57.4%) miRNAs were up‐regulated and 190 (37.8%) lncRNAs, 787 (55.5%) mRNAs and 80 (42.6%) miRNAs were down‐regulated in CRC patients compared with normal tissue samples (Figure S1).

### 3.2 Gene ontology and pathway analysis of differentially expressed genes

**Figure 2:**
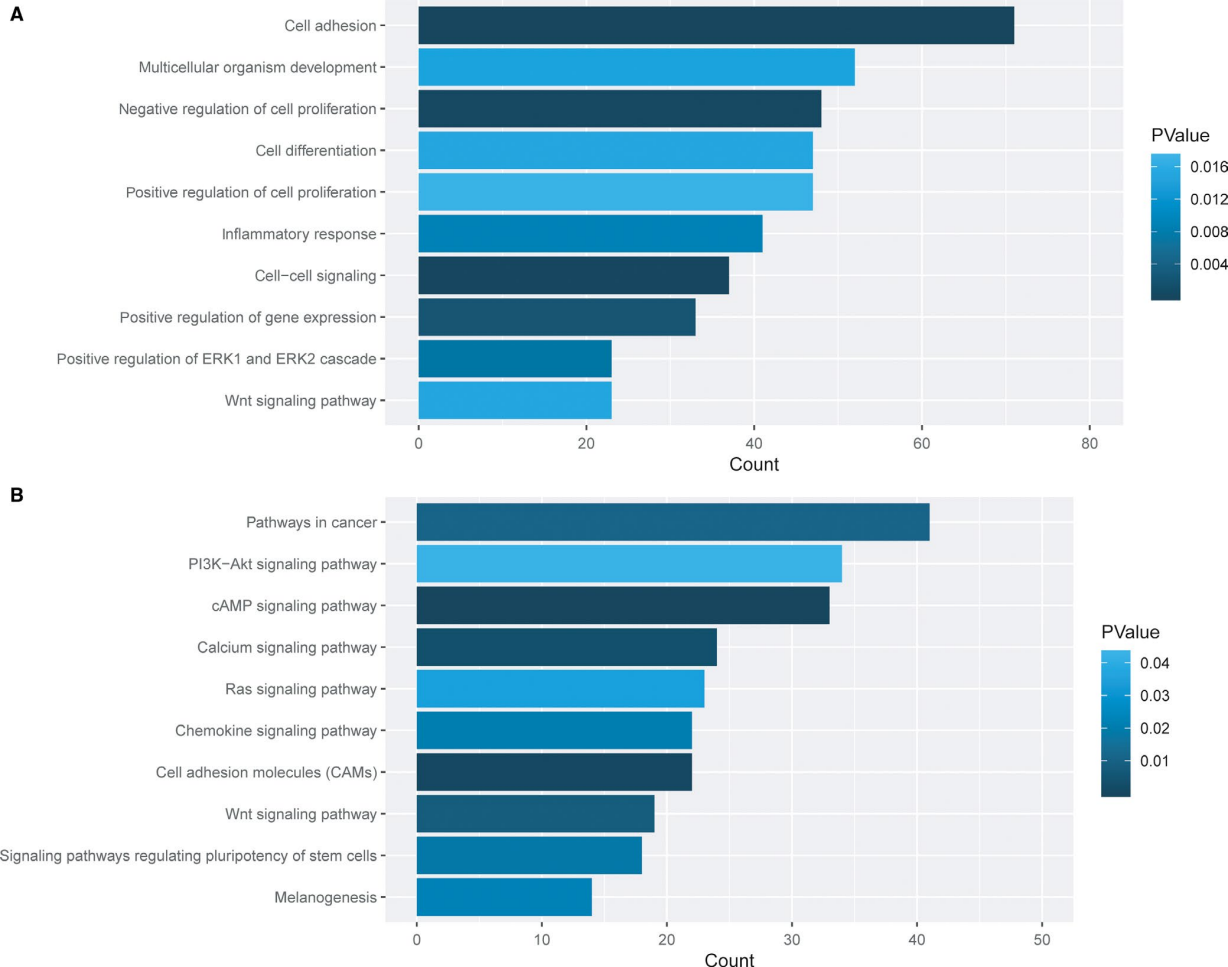
Biological function and pathway analysis of differentially expressed mRNAs. A, The top 10 significant functional annotations in the GO biological process. B, The top 10 significant functional annotations in the pathway

To explore the potential functional implication of the 1419 DEmRNAs, functional enrichment analysis of GO and KEGG was performed for DEmRNAs. A total of 291 enriched GO terms in the Biological Process (BP) were identified after GO analysis. The top 10 significantly enriched BPs are shown in Figure 2A. We found that the DERNAs were mainly enriched in cancer‐related biological processes, such as ‘Wnt signalling pathway’, ‘positive regulation of ERK1 and ERK2 cascade’, ‘positive regulation of gene expression’. Additionally, KEGG pathway analysis showed that 58 pathways were significantly enriched. The top 10 significantly enriched pathways are shown in Figure 2B. Among these pathways, the ‘Pathways in cancer’, ‘PI3K‐Akt signalling pathway’, ‘Ras signalling pathway’ and ‘Wnt signalling pathway’ are closely correlated with the carcinogenesis and development of CRC.

### 3.3 Development of a ceRNA regulatory network in CRC

To elucidate the regulatory mechanism of CRC, a lncRNA‐miRNA‐mRNA related ceRNA network of CRC was developed according the above results. First, we searched the 503 DElncRNAs in the StarBase and miRcode database and 1146 interactive lncRNAs‐miRNAs pairs were found. Among these pairs, 24 DEmiRNAs were confirmed to interact with 46 DElncRNAs. Following this, we predicted that 1300 mRNAs were targeted by these 24 DEmiRNAs in all three target‐predicting databases. The 1300 targeted mRNAs were further intersected with the 1419 DEmRNAs and mRNAs not included in DEmRNAs were excluded. The results show that 45 mRNAs were finally included in the development of the network.

**Figure 3:**
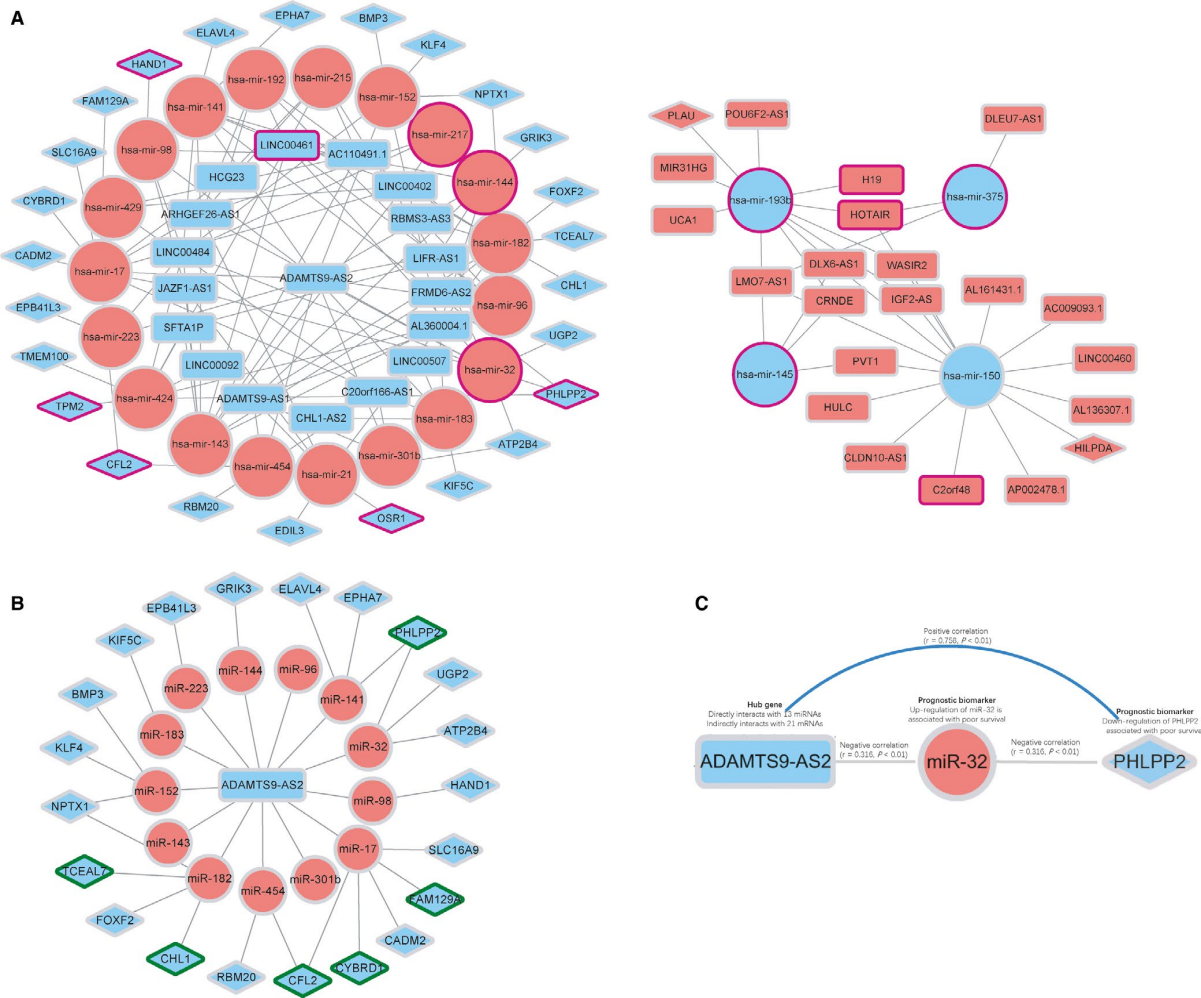
A, The overall lncRNA‐miRNA‐mRNA ceRNA network in colorectal cancer. B, The sub‐network centre on ADAMTS9‐AS2. C, The ADAMTS9‐AS2/miR‐32/PHLPP2 ceRNA regulatory axis. The red nodes represent high expression, while the blue nodes represent low expression. miRNAs, lncRNAs and mRNA are represented by ellipse, round rectangle and diamonds respectively. Purple borders surrounding the nodes indicate prognostic significance. Green borders surrounding the nodes indicate good correlation with ADAMTS9‐AS2. Grey edges indicate interactions between RNAs

The lncRNA‐miRNA and miRNA‐mRNA relationship pairs (Tables S2 and S3) were combined into the ceRNA network following the pattern of negative regulation. The links between positively coexpressed lncRNA‐miRNA pairs and miRNA‐mRNA pairs were discarded. Finally, we constructed the ceRNA regulatory network of CRC comprised of 125 edges among 38 DElncRNAs, 23 DEmiRNAs and 27 DEmRNAs (Figure 3A**)**.

Moreover, we found that the lncRNA ADAMTS9‐AS2 may play the important role of hub gene in the ceRNA network. ADAMTS9‐AS2 interacted with 12 miRNAs (miR‐143, miR‐223, miR‐98 miR‐152, miR‐182, miR‐183, miR‐141, miR‐32, miR‐17, miR‐454, miR‐96, miR‐144 and miR‐301b) and indirectly interacted with 21 miRNA‐targeted mRNAs (RBM20, UGP2, ATP2B4, TCEAL7, FOXF2, FAM129A, PHLPP2, CFL2, CYBRD1, KLF4, BMP3, EPHA7, HAND1, PHLPP2, EPB41L3, KIF5C, CHL1, SLC16A9, NPTX1, GRIK3 and ELAVL4) in this network. We speculated that ADAMTS9‐AS2 might greatly contribute to the carcinogenesis of CRC (Figure 3B). Hierarchical cluster heatmaps of the mRNAs, lncRNAs and miRNAs that involved in the ceRNA are shown in Figure S2.

### 3.4 Prognostic characteristics of RNAs in the regulatory network

**Figure 4:**
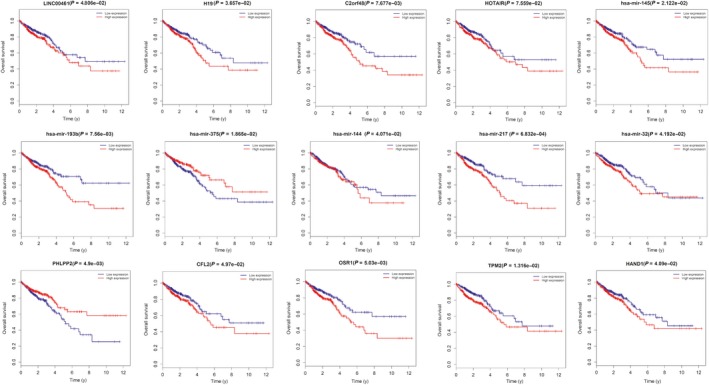
Kaplan‐Meier curve of RNAs that are significantly associated with overall survival in colorectal cancer patients

Kaplan‐Meier survival analysis was used to investigate the correlation between expression levels of DERNAs in the ceRNA network and the survival outcome of CRC patients. The results show that four lncRNAs (LINC00461, H19, C2orf48 and HOTAIR), six miRNAs (miR‐145, miR‐193b, miR‐375, miR‐144, miR‐217 and miR‐32) and five mRNAs (PHLPP2, CFL2, OSR1, TPM2 and HAND1) were significantly correlated with overall survival (*P* < 0.05) (Figure 4).

### 3.5 Interaction between lncRNA and mRNA from the ceRNA Network

**Figure 5:**
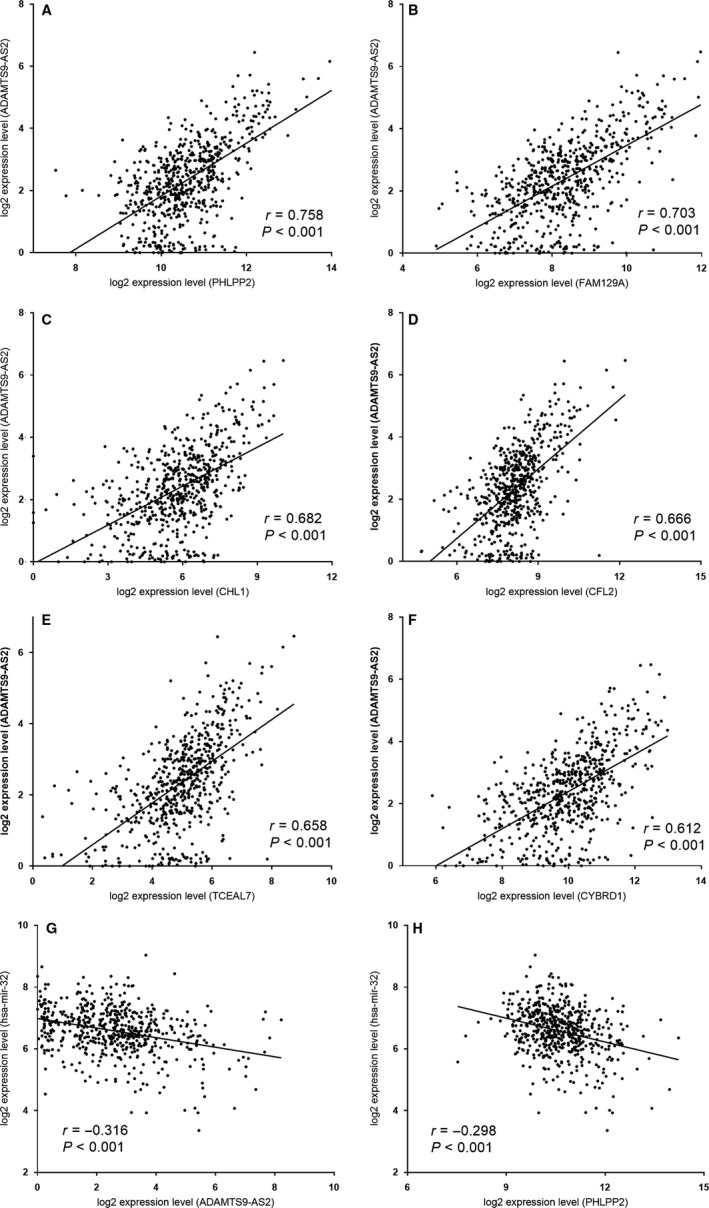
Pearson's correlation analysis on the correlation between the expression levels of ADAMTS9‐AS2 and mRNAs in ceRNA network (A‐F); and correlation between hsa‐mir‐32 and ADAMTS9‐AS2 and PHLPP2 (G and H). The ‘*r*’ indicates correlation coefficient

According to the ceRNA theory, lncRNAs could indirectly interact with mRNAs in the post‐transcriptional regulatory network. To validate whether the network fit in with ceRNA theory, we conducted analysis on the correlation between expression levels of lncRNA and those of mRNAs. It showed that there was a strong positive correlation between expression levels of ceRNAs (Table S4). For instance, ADAMTS9‐AS2 positively correlated with PHLPP2 (*r* = 0.758, *P* = 2.21e‐112), FAM129A (*r* = 0.703, *P* = 3.32e‐90), CHL1 (*r* = 0.682, *P* = 9.99e‐83), CFL2 (*r* = 0.666, *P* = 1.14e‐77), TCEAL7 (*r* = 0.658, *P* = 3.03e‐75) and CYBRD1 (*r* = 0.612, *P* = 1.3e‐62). In addition, hsa‐mir‐32 negatively interacted with lncRNA ADAMTS9‐AS2 (*r* = ‐ 0.316, *P* = 3.57e‐9) as well as PHLPP2 (*r* = −0.298, *P* = 3.32e‐2) (Figure 5).

### 3.6 Experimental validation

**Figure 6:**
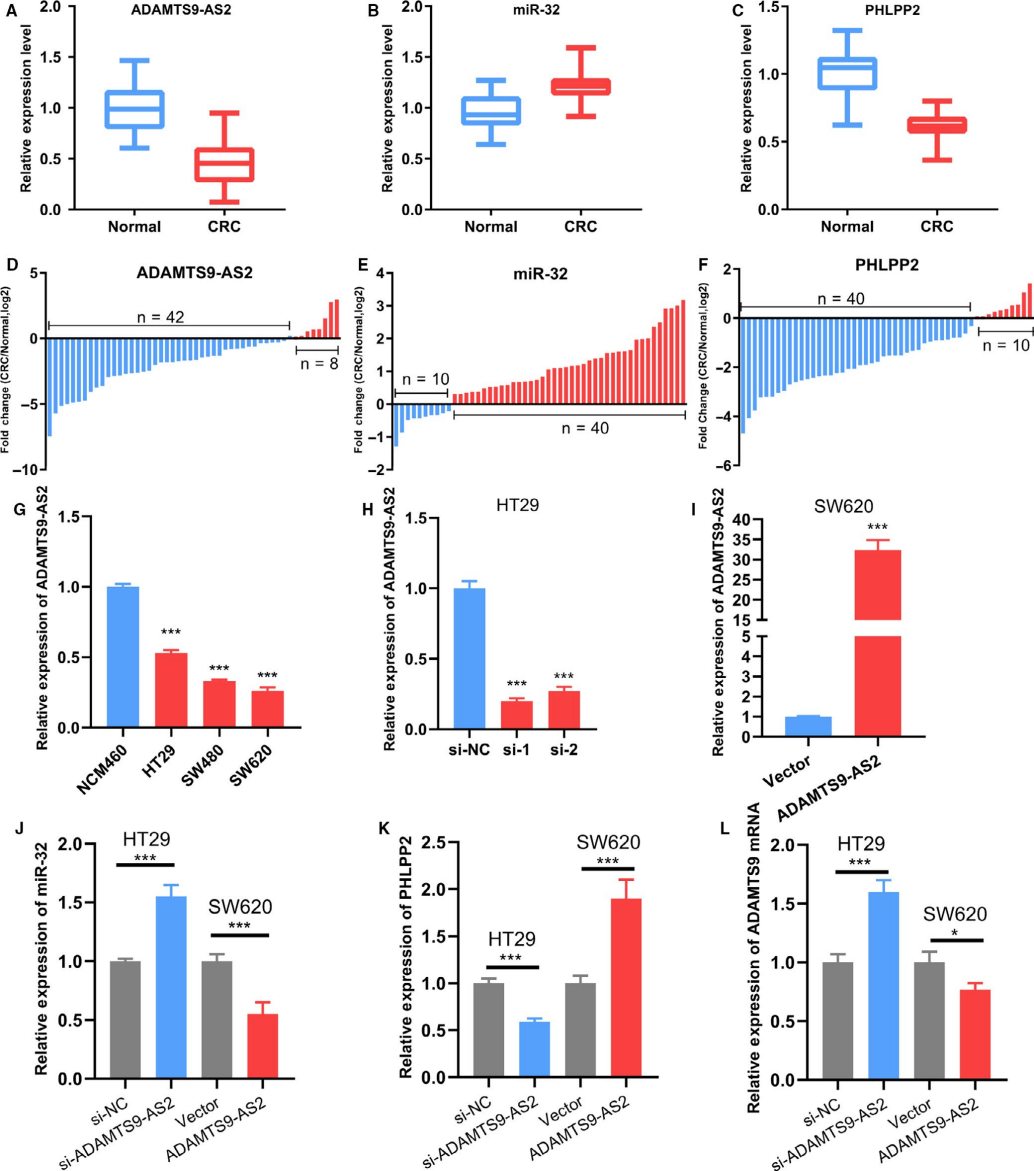
The expression levels of ADAMTS9‐AS2, miR‐32 and PHLPP2 in 50 pairs of CRC tissues and adjacent normal tissues and colon cancer cell lines measured by qRT‐PCR. (A‐E) ADAMTS9‐AS2 and PHLPP2 expressions significantly decrease in CRC tissues, while miR‐32 expression significantly increases in CRC compared with normal tissues. (G) ADAMTS9‐AS2 was down‐regulated in colon cancer cell lines compared with NCM460 cells. (H‐I) The effects of knockdown and overexpression of ADAMTS9‐AS2 were measured by qRT‐PCT in HT29 and SW620 cells. (J‐L) The expression levels of miR‐32, PHLPP2 and ADAMTS9 after knockdown or overexpression of ADAMTS9‐AS2 in HT29 or SW620 cells. (*P < 0.05, **P < 0.01, ***P < 0.001)

The expression levels of ADAMTS9‐AS2, miR‐32 and PHLPP2 were investigated in 54 paired CRC and adjacent non‐tumour tissue samples by qRT‐PCR (Figure 6A‐F). ADAMTS9‐AS2 and PHLPP2 expressions were down‐regulated in 84% (42/50) and 80% (40/50) of CRC tissues respectively and miR‐32 was up‐regulated in 80% (40/50) compared with that in adjacent normal tissues. Down‐regulated expression of ADAMTS9‐AS2 was correlated with the advanced TNM stage (*P* = 0.010) and poor histologic differentiation (*P* = 0.012) (Table 1). Overexpression of miR‐32 was associated with larger tumour size (*P* = 0.012) and down‐regulated PHLPP2 was correlated with the advanced TNM stage (*P* = 0.002).

**Table 1:** Correlation between ADAMTS‐AS2, miR‐32 and PHLPP2 mRNA expression levels and the clinicopathological parameters of 50 CRC patients

Clinicopathological parameters	Number of cases	ADAMTS9‐AS2 expression		*P* value	miR‐32 expression		*P* value	PHLPP2 expression		*P* value
		High	Low		High	Low		High	Low	
Gender				0.758			0.123			0.123
Male	35	17	18		15	20		20	15	
Female	15	8	7		10	5		5	10	
Age (y)				0.564			1.000			0.083
≥65	30	16	14		15	15		12	18	
<65	20	9	11		10	10		13	7	
Tumour size				0.529			0.012*			0.208
>5 cm	14	8	6		11	3		9	5	
≤5 cm	36	17	19		14	22		16	20	
Histologic differentiation				0.012*			0.208			0.059
Well or moderate	36	22	14		16	20		15	21	
Poor	14	3	11		9	5		10	4	
TNM stage				0.010*			0.152			0.002**
I‐II	29	19	10		12	17		20	9	
III‐IV	21	6	15		13	8		5	16	
Serum CEA level				0.785			0.396			0.157
>5 ng/ml	25	12	13		14	11		10	15	
≤5 ng/ml	25	13	12		11	14		15	10	
Lymphovascular invasion				0.346			1.000			0.346
Negative	45	21	24		22	23		21	24	
Positive	5	4	1		3	2		4	1	
Perineural invasion				1.000			0.470			0.470
Negative	48	24	24		25	23		23	25	
Positive	2	1	1		0	2		2	0	

Abbreviations: CEA carcinoembryonic antigen; TNM tumour‐node‐metastasis stage.

* *P* < 0.05;

** *P* < 0.01.

Furthermore, ADAMTS9‐AS2 was underexpressed in three colon cancer cell lines (HT29, SW480 and SW620), when compared to the normal colon cell line (NCM460) (Figure 6G). HT29 cells demonstrated a relatively higher expression level of ADAMTS9‐AS2, contrary to the SW620 cells which exhibited a relatively lower expression level. Therefore, HT29 cells were selected for ADAMTS9‐AS2 knockdown and SW620 cells were chosen for overexpression. The effects of ADAMTS9‐AS2 knockdown and overexpression were determined by qRT‐PCR (Figure 6H‐I).

Then the expression levels of miR‐32, PHLPP2 and ADAMTS9 mRNA after ADAMTS9‐AS2 knockdown or overexpression were measured by qRT‐PCR. The miR‐32 expression significantly increased after ADAMTS9‐AS2 knockdown in HT29 cells and decreased after ADAMTS9‐AS2 overexpression in SW620 cells (Figure 6J). On the contrary, the PHLPP2 expression was inhibited in HT29 cells with ADAMTS9‐AS2 knockdown and was promoted in SW620 cells with ADAMTS9‐AS2 overexpression (Figure 6K). Notably, the mRNA expression level of ADAMTS9 was significantly increased after ADAMTS9‐AS2 knockdown and decreased after ADAMTS9‐AS2 overexpression (Figure 6L).

## 4 DISCUSSION

As shown in the present study, differentially expressed mRNAs, lncRNAs and miRNAs were identified in tumour samples compared with adjacent non‐tumour samples from TCGA‐COAD and READ database respectively. To improve the reliability, the CRC‐specific RNAs were defined as the intersected DERNAs of the two databases. Then, according to ceRNA theory, we constructed a lncRNA‐miRNA‐mRNA regulatory network by integrated bioinformatics analysis. The functional annotations of targeted genes were studied by GO and Pathway analysis. Furthermore, these RNAs were investigated for their relationship with overall survival. Subsequently, the correlation between expression levels and the clinicopathological features were validated in clinical samples by qRT‐PCR. The regulatory effects in the ADAMTS9‐AS2/miR‐32/PHLPP2 axis was proved by knockdown or overexpression of ADAMTS9‐AS2 in colon cancer cell lines. Our study may provide a comprehensive perspective of the potential mechanisms of gene interaction and regulation in CRC.

Among the ceRNA network, the lncRNA ADAMTS9‐AS2 is the most notable gene because it has the highest degree of connectivity to other nodes, although its relationship with survival was not significant, we assumed that it was a hub gene that plays critical roles in the network. Then we extracted a sub‐network that centred on ADAMTS9‐AS2 for further analysis. The results showed that ADAMTS9‐AS2 directly interacted with 13 miRNAs and indirectly interacted with 21 miRNA‐targeted mRNAs in this network (Figure 3B).

By investigating the sub‐network, we found that overexpression of three miRNAs (hsa‐mir‐32, hsa‐mir‐217 and hsa‐mir‐144) was associated with unfavourable prognosis. Further analysis indicated that there was an interaction between hsa‐mir‐32 and mRNA PHLPP2. Our results revealed that PHLPP2 was significantly down‐regulated in the CRC tissues in comparison with adjacent non‐tumour tissue and the low expression of PHLPP2 was significantly related to poor survival.

To further explore the interaction, Pearson's correlation analysis was conducted among those RNAs. It revealed that there were negative correlations between the expression of miR‐32 and the expression of ADAMTS9‐AS2(*r *= −0.316, *P* < 0.01) as well as PHLPP2 (*r *= −0.298, *P* < 0.01) in CRC samples. Furthermore, a potential positive interaction between ADAMTS9‐AS2 and PHLPP2 expression levels was detected (*r* = 0.758, *P* < 0.01). ADAMTS9‐AS2 and PHLPP2 expressions were significantly down‐regulated in CRC specimens, while miR‐32 was up‐regulated in the CRC tissues. These findings indicate that lncRNA ADAMTS9‐AS2 may regulate the expression of PHLPP2 by negatively interacting with hsa‐mir‐32. These regulatory patterns perfectly conformed to the ‘ceRNA theory’ and these findings were validated in clinical samples and cell lines by qRT‐PCR. Therefore, we have been suggested that the ceRNA axis ADAMTS9‐AS2/miR‐32/PHLPP2 plays important roles in molecular regulation mechanism of CRC (Figure 3C).

The lncRNA ADAMTS9‐AS2 is a novel tumour suppressor and is the antisense transcript of ADAMTS9. However, relevant research on ADAMTS9‐AS2 in cancer is still limited. Previous researches have reported that ADAMTS9‐AS2 was down‐regulated in colorectal cancer, clear cell renal cell carcinoma, lung cancer and glioma.^23^, ^24^, ^25^, ^26^, ^27^ Moreover, ADAMTS9‐AS2 expression was negatively correlated with the survival of these cancers. miR‐32 is a tumour‐associated microRNA, but its role in cancer is contradictory. Fu et al found that up‐regulated miR‐32‐5p activated the PI3K/Akt pathway and led to drug resistance by promoting epithelial‐mesenchymal transition and angiogenesis.^28^ It has been reported that high expression of miR‐32‐5p was positively associated with poor survival outcome of hepatocellular carcinoma^28^ and gastric cancer.^29^ However, Wang et al reported that miR‐32‐5p significantly inhibited metastasis of clear cell renal cell carcinoma.^30^ PHLPP2 is a phosphatase‐regulating AKT(S473) phosphorylation and mTORC2. PHLPP2 acted as tumour suppressors in various cancers by their capability of blocking growth factor‐induced signalling pathway in cancer cells.^31^, ^32^, ^33^ Xia et al reported that PHLPP2‐knockdown in miR‐32‐transfected breast cancer cells promotes cell proliferation, indicating that PHLPP2 down‐regulation is an important part for miR‐32‐induced cell proliferation.^34^

To summary, a ceRNA regulatory network was successfully developed by identification of cancer‐specific lncRNAs, miRNAs and mRNAs from large‐scale CRC samples in TCGA database. We propose that the regulatory network centred on ADAMTS9‐AS2 may play a critical part in the carcinogenesis of CRC. Our study highlighted a novel ceRNA mechanism in which ADAMTS9‐AS2 sponging miR‐32 to regulate the expression of PHLPP2. However, the ADAMTS9‐AS2/miR‐32/PHLPP2 regulatory axis requires further studies to fully elucidate their biological functions and to confirm the molecular mechanisms.

## CONFLICT OF INTEREST

The authors declare that they have no competing interests.
