# Supplementary figures and images for: Identification and development of long non‐coding RNA‐associated regulatory network in colorectal cancer
Source: J Cell Mol Med. 2019 May 29;23(8):5200–10. doi: 10.1111/jcmm.14395 (PMC6653593; doi:10.1111/jcmm.14395)

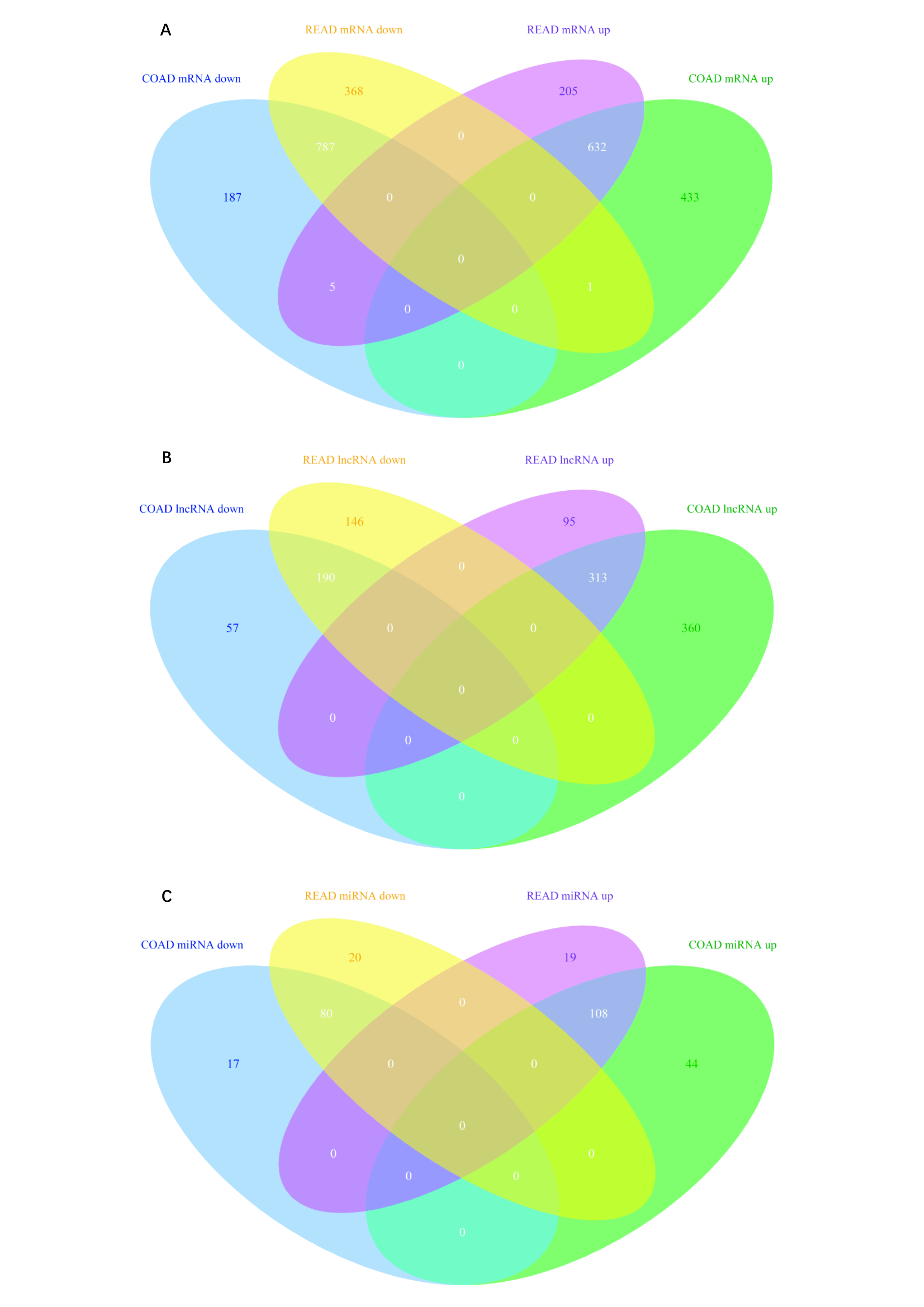

Supplement: Supplementary file 1 [file JCMM-23-5200-s001.tif]

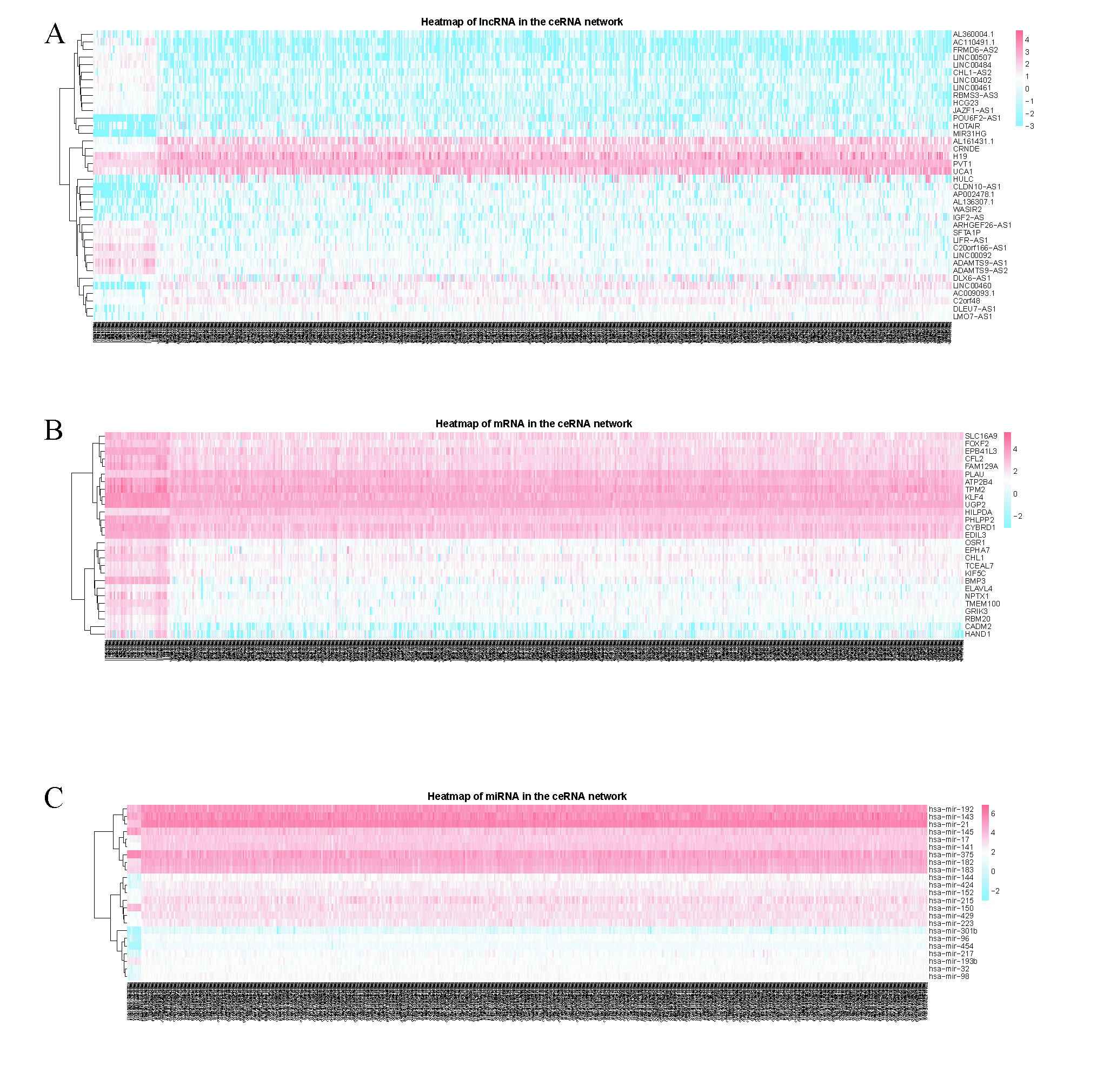

Supplement: Supplementary file 2 [file JCMM-23-5200-s002.tif]
