# Supplementary material for: Identification and development of long non‐coding RNA‐associated regulatory network in colorectal cancer
Source: J Cell Mol Med. 2019 May 29;23(8):5200–10. doi: 10.1111/jcmm.14395 (PMC6653593; doi:10.1111/jcmm.14395)
Supplement: Supplementary file 4 [file JCMM-23-5200-s004.docx]

## Supplementary files

**TABLE S1** Accession numbers of tumor and normal tissue samples in TCGA-COAD and TCGA-READ datasets (uploaded seperately)

| **TABLE S2**. Interaction between lncRNA and miRNA in the ceRNA network | |
| --- | --- |
| lncRNA | miRNA |
| AC009093.1 | miR-150 |
| AC110491.1 | miR-215, miR-143, miR-192, miR-182, miR-98, miR-141, miR-429 |
| ADAMTS9-AS1 | miR-144, miR-182, miR-21, miR-454, miR-96, miR-301b |
| ADAMTS9-AS2 | miR-141, miR-96, miR-144, miR-152, miR-182, miR-183, miR-223, miR-32, miR-17, miR-454, miR-143, miR-98, miR-301b |
| AL136307.1 | miR-150 |
| AL161431.1 | miR-150 |
| AL360004.1 | miR-96, miR-144, miR-152, miR-21, miR-183, miR-182, miR-424, miR-143 |
| AP002478.1 | miR-150 |
| ARHGEF26-AS1 | miR-215, miR-143, miR-217, miR-192, miR-21, miR-17, miR-32, miR-141 |
| C20orf166-AS1 | miR-17, miR-183, miR-454, miR-301b, miR-429 |
| C2orf48 | miR-150 |
| CHL1-AS2 | miR-183 |
| CLDN10-AS1 | miR-150 |
| CRNDE | miR-193b, miR-145 |
| DLEU7-AS1 | miR-375 |
| DLX6-AS1 | miR-150, miR-193b, miR-145 |
| FRMD6-AS2 | miR-96, miR-182, miR-143 |
| H19 | miR-193b |
| HCG23 | miR-215, miR-192, miR-17 |
| HOTAIR | miR-150, miR-193b, miR-375 |
| HULC | miR-150 |
| IGF2-AS | miR-150, miR-193b |
| JAZF1-AS1 | miR-143, miR-17, miR-21, miR-32, miR-98 |
| LIFR-AS1 | miR-144, miR-182, miR-192, miR-32, miR-96, miR-215 |
| LINC00092 | miR-424 |
| LINC00402 | miR-17, miR-143, miR-141, miR-217, miR-182, miR-429 |
| LINC00460 | miR-150 |
| LINC00461 | miR-141, miR-215, miR-192, miR-144, miR-32, miR-424, miR-96, miR-143 |
| LINC00484 | miR-223, miR-143, miR-152, miR-217, miR-141, miR-32, miR-424, miR-98 |
| LINC00507 | miR-183 |
| LMO7-AS1 | miR-375, miR-193b, miR-150, miR-145 |
| MIR31HG | miR-193b |
| POU6F2-AS1 | miR-193b |
| PVT1 | miR-150, miR-145 |
| RBMS3-AS3 | miR-182, miR-96 |
| SFTA1P | miR-143, miR-182, miR-424 |
| UCA1 | miR-193b |
| WASIR2 | miR-150, miR-193b |

| **TABLE S3**. Interaction between miRNA and mRNA in the ceRNA network | |
| --- | --- |
| miRNA | mRNA |
| hsa-mir-17 | SLC16A9, CYBRD1, CFL2, PHLPP2, FAM129A |
| hsa-mir-21 | EDIL3, OSR1, ATP2B4 |
| hsa-mir-32 | PHLPP2, ATP2B4, UGP2 |
| hsa-mir-98 | HAND1 |
| hsa-mir-141 | ELAVL4, EPHA7, PHLPP2 |
| hsa-mir-144 | GRIK3 |
| hsa-mir-150 | HILPDA |
| hsa-mir-152 | NPTX1, BMP3, KLF4 |
| hsa-mir-182 | CHL1, FOXF2, TCEAL7, NPTX1 |
| hsa-mir-183 | KIF5C |
| hsa-mir-193b | PLAU |
| hsa-mir-223 | EPB41L3 |
| hsa-mir-424 | PHLPP2, TPM2, TMEM100 |

| **TABLE S4**. Correlation analysis of the relationship between lncRNA and mRNA | | | |
| --- | --- | --- | --- |
| lncRNA | mRNA | P value | R |
| AC110491.1 | PHLPP2 | 6.66E-109 | 0.750 |
| AC110491.1 | TPM2 | 1.78E-102 | 0.735 |
| AC110491.1 | CFL2 | 3.77E-98 | 0.724 |
| AC110491.1 | OSR1 | 1.76E-82 | 0.681 |
| AC110491.1 | FAM129A | 2.70E-72 | 0.648 |
| ADAMTS9-AS1 | PHLPP2 | 2.67E-91 | 0.706 |
| ADAMTS9-AS1 | CFL2 | 1.43E-76 | 0.662 |
| ADAMTS9-AS1 | FAM129A | 1.40E-71 | 0.645 |
| ADAMTS9-AS2 | PHLPP2 | 2.21E-112 | 0.758 |
| ADAMTS9-AS2 | FAM129A | 3.32E-90 | 0.703 |
| ADAMTS9-AS2 | CHL1 | 9.99E-83 | 0.682 |
| ADAMTS9-AS2 | CFL2 | 1.14E-77 | 0.666 |
| ADAMTS9-AS2 | TCEAL7 | 3.03E-75 | 0.658 |
| ADAMTS9-AS2 | CYBRD1 | 1.30E-62 | 0.612 |
| AL360004.1 | ATP2B4 | 8.57E-61 | 0.605 |
| C20orf166-AS1 | NPTX1 | 6.20E-72 | 0.646 |
| C20orf166-AS1 | TPM2 | 1.25E-70 | 0.642 |
| C20orf166-AS1 | ATP2B4 | 2.24E-61 | 0.607 |
| JAZF1-AS1 | CFL2 | 4.81E-61 | 0.606 |
| JAZF1-AS1 | ATP2B4 | 1.18E-60 | 0.604 |

**Figure S1** Venn diagrams selecting intersected RNAs that up-regulated or down-regulated in COAD and READ databases. (A) Intersection in mRNA; (B) lncRNA; (C) miRNA

**Figure S2** Hierarchical cluster heatmap of the RNAs included in the ceRNA network. Each row represents a transcript and each column indicates one sample, the color from red to blue shows the dysregulation of expression from high to low. (A)lncRNA; (B)mRNA; (C)miRNA.
